# Physics-Informed Neural Networks for the Condition Monitoring of Rotating Shafts

**DOI:** 10.3390/s24010207

**Published:** 2023-12-29

**Authors:** Marc Parziale, Luca Lomazzi, Marco Giglio, Francesco Cadini

**Affiliations:** Department of Mechanical Engineering, Politecnico di Milano, Via La Masa 1, 20156 Milan, Italy; marc.parziale@polimi.it (M.P.); luca.lomazzi@polimi.it (L.L.); marco.giglio@polimi.it (M.G.)

**Keywords:** condition monitoring, rotating shaft, physics-informed neural network, parameters estimation

## Abstract

Condition monitoring of rotating shafts is essential for ensuring the reliability and optimal performance of machinery in diverse industries. In this context, as industrial systems become increasingly complex, the need for efficient data processing techniques is paramount. Deep learning has emerged as a dominant approach due to its capacity to capture intricate data patterns and relationships. However, a prevalent challenge lies in the black-box nature of many deep learning algorithms, which often operate without adhering to the underlying physical characteristics intrinsic to the studied phenomena. To address this limitation and enhance the fusion of data-driven methodologies with the fundamental physics of the system under study, this paper leverages physics-informed neural networks (PINNs). Specifically, a simple but realistic numerical case study of an extended Jeffcott rotor model, encompassing damping effects and anisotropic supports for a more comprehensive modelling, is considered. PINNs are used for the estimation of five parameters that characterize the health state of the system. These parameters encompass the radial and angular position of the static unbalance due to the disk installed on the shaft, the stiffness along the principal axes of elasticity, and the non-rotating damping coefficient. The estimation is conducted solely by exploiting the displacement signals from the centre of the disk and, to showcase the efficacy and precision provided by this novel methodology, various scenarios involving different constant rotational speeds are examined. Additionally, the impact of noisy input data is also taken into account within the analysis and the performance is compared to that of traditional optimization algorithms used for parameters estimation.

## 1. Introduction

Rotating shafts are elements of engineering systems that play a paramount role in the transmission of power, encompassing speed and torque, from one point to another [1,2]. They are typically designed to endure substantial loads and to operate at high velocities, underscoring the need for precise alignment, equilibrium, and freedom from imperfections. These considerations are significant not only for enhancing the overall system performance, but also for improving its safety and reliability [3,4]. To attain this objective, the practice of condition monitoring (CM) for rotating shafts allows continuously evaluating the shaft condition and performance and detecting any indications of malfunction or deterioration [5,6]. Through the application of CM methodologies, potential issues can be promptly identified, and maintenance or repair actions can be driven to prevent accidents. This approach has shown to effectively mitigate the risk of unexpected downtime, reinforcing the overall system reliability [7,8]. Furthermore, the widespread and cost-effective availability of sensors has revolutionized the acquisition of diagnostic signals, such as accelerations, strains, and elastic waves [9]. However, the availability of big data is itself a new layer of complexity, especially in the realm of signal processing. That is, the rapid expansion of the amount of acquired data has introduced the need for (i) improved hardware and software performance, and (ii) developing tools to deal with confounding factors, including those unrelated to the system health state, such as environmental and operational conditions [10,11].

To tackle these challenges, deep learning has stood as a pivotal technological advancement in the CM of rotating machines, offering multifaced contributions of significant importance [12]. This approach has excelled in automatically extracting intricate patterns and features from raw sensor data, enhancing the precision and reliability of fault detection and anomaly characterization. As an example, the work in [13] applied deep learning to enhance wind turbine CM, addressing the data surge from increased wind farm units. By combining convolutional neural networks (CNNs) [14] and recurrent neural networks (RNNs) [15], it efficiently extracted features, reduced dimensionality, and provided effective CM, offering both real-time unit state checks and early warning capability, even amidst accidental parameter changes. In [16], a CM model based on CNNs for automatic fault detection in rotating equipment was developed. The model, utilizing data from a single vibration sensor on the motor-drive end bearing, achieved accuracies of 99.58% and 97.3% when applied to two different databases under controlled ambient conditions. Another example was presented in [17], where the authors proposed a novel deep learning algorithm for detecting rotor unbalance in industrial machinery. The algorithm, extracting important vibration signatures such as fast Fourier transform (FFT) and short-time Fourier transform (STFT), combined the depth of ResNet [18] and the feature extraction capability of CNN. This hybrid approach surpassed the performance of both individual models. The study involved two analyses: binary detection of balanced vs. unbalanced cases and multilevel detection of the degree of unbalance. The work in [19] addressed planetary gearbox fault detection by representing baseline vibration signals using the varying index coefficient autoregression (VICAR) model. The authors proposed a modified VICAR (MVICAR) model to effectively incorporate rotating speed into the representation while maintaining nonlinear modeling capacity. Experimental results demonstrated the superiority of the MVICAR model over autoencoders, expanded VICAR (EVICAR), and linear parameter-varying autoregression models in planetary gearbox fault detection. In [20], a semi-supervised fault diagnosis approach for wind turbines was introduced. The method utilized a deep neural network with adversarial learning and incorporated a metric-guided feature enhancement technique. Despite having a limited number of annotated samples, the methodology exhibited superior fault diagnosis accuracy in experiments conducted on a wind turbine fault dataset.

However, in the context of CM, the developed methods have predominantly relied on black-box deep learning algorithms, lacking transparency in how input data are processed and whether the network behavior aligns with the physics of the problem [21]. Existing approaches to address this issue involve either post-training explainability algorithms or more intricate physics-based deep learning models. The former, while debunking network behavior, fails to provide evidence of adherence to physical laws [22,23,24,25]. On the other hand, the latter ensures predictions align with the physics by incorporating regularization terms representing known physical laws during training. These terms are integrated into the network loss function, specifically at the stage where it quantifies the disparity between predicted and actual outcomes. This critical addition serves to guide the neural network towards solutions that not only capture intricate patterns from data but also adhere rigorously to the established physical laws, enhancing the reliability and interpretability of physics-informed neural network (PINN) predictions. The regularization terms act as foundational constraints, influencing the network learning to prioritize solutions that respect the governing physics throughout the training iterations. Moreover, physics-informed algorithms offer a distinct advantage by providing accurate predictions even in the presence of scarce data, a capability not shared by traditional deep learning methods. Notably, physics-informed algorithms are versatile tools applicable in various contexts, including data-driven solutions for partial differential equations, discovery of physical laws, and parameter estimation [26,27,28]. However, within the CM domain, few contributions have integrated physical knowledge effectively into the training process of deep learning models. In [29], the authors introduced a novel approach for fault detection in gearboxes using long-short term memory (LSTM) neural networks. Given a lack of data from faulty states, the authors proposed a physics-informed hyperparameter selection strategy for LSTM identification, emphasizing maximizing the discrepancy between healthy and physics-informed faulty states. Case studies on detecting gear tooth crack and tooth wear demonstrated that the approach outperformed traditional methods based on minimizing validation mean squared error (VAMSE). The work in [30] presented a physics-informed deep learning method for bearing fault detection that combined a threshold model and a CNN. The approach was validated using data from bearings on an agricultural machine and a laboratory test stand in the Case Western Reserve University Bearing Data Centre. In [31], a method for identifying unbalance faults in rotary systems using physics-guided neural networks (PGNNs) was proposed. The approach involved the use of a standard neural network to localize the nodal position of the experimental fault, followed by PGNN to quantify the unbalance magnitude and phase angle. Instead, the work in [32] introduced a novel physics-informed convolution long-short-term memory (LSTM-CNN) network for rotor unbalance and shaft cracks detection and localization. In particular, the physics were taken into account through the construction of a neural network model which mimicked a finite element (FE) resolution of the problem.

To the best of the authors’ knowledge, still no efforts have been made for the direct estimation of multiple parameters characterizing the health state of a rotating shaft system by leveraging PINNs. In this work, PINNs are utilized to estimate critical health state parameters in a simple but realistic numerical case of an extended Jeffcott rotor model. This model incorporates damping effects and anisotropic supports for a more comprehensive representation. The parameters under consideration include the radial and angular position of the static unbalance caused by the disk on the shaft, stiffness along the principal axes of elasticity, and the non-rotating damping coefficient. The estimation is exclusively based on the displacement signals from the disk centre. Note that this estimation not only optimizes the performance of machineries, enhancing efficiency and reliability, but also enables predictive maintenance by identifying potential faults early on. To highlight the effectiveness and precision of the proposed methodology, various scenarios with different constant rotational speeds are examined, and the performance is compared to that of traditional optimization algorithms used for parameters estimation. Furthermore, the analysis accounts for the impact of noisy input data. It is important to note that the proposed work presents a proof of concept, demonstrating the effectiveness of the proposed methodology through simulation experiments in a controlled environment. The transition from simulations to real-world applications is highlighted, emphasizing the commitment to practicality. Subsequent efforts will focus on rigorous experimental validation and testing on more complex systems to enhance the approach versatility and robustness.

The main innovation of this work lies in integrating established physical knowledge, describing the fundamental dynamics of rotating shaft systems, into the neural network training process. This incorporation serves to guide the training, enhancing the robustness and reliability in the system health state parameter estimation. Furthermore, the estimation relies exclusively on raw time-domain displacements at the disk centre, minimizing the requirement for numerous sensors and simplifying the overall preprocessing steps.

The paper is organised as follows: Section 2 offers a brief overview of the necessary theoretical foundations about PINNs for parameter estimation; Section 3 shortly presents the case study and then shows in detail the implementation and the results of the PINN for the system health state characterization. Finally, Section 4 provides some concluding remarks.

## 2. Methodology

The proposed framework hinges upon the use of PINNs to estimate the unknown parameters characterizing the dynamics of a rotating shaft system. The innovative aspect in this methodology stems from the tailored and specific application of PINNs, addressing the challenges and requirements associated with accurately estimating health parameters in the context of rotating shaft systems. Notably, PINNs represent deep learning tools that combine NNs with the system governing equations, and are particularly useful when data might be limited or noisy, and where the underlying physics of the problem is well understood [26].

Assume that a generic physical system is governed by the n-th order ordinary differential equations (ODEs) shown in Equation (1):

(1) $$u^{\left(n\right)}=F(t,u,u^{\prime },u^{\prime \prime },\ldots ,u^{\left(n-1\right)},\lambda )$$

where t refers to the system independent variable, $u=[u_{1}\left(t\right),u_{2}\left(t\right),\ldots ,u_{p}\left(t\right)]$ denotes the state vector consisting of p components defined in the domain $\left[t_{0},t_{f}\right]$, and $\lambda =[\lambda_{1},\lambda_{2},\ldots ,\lambda_{k}]$ represents the vector made of the k unknown parameters describing the system state. Subsequently, considering the NN universal approximation theorem [33], an NN can be exploited to obtain an approximation $\hat{u}=N(W,b,\lambda )$ of the state vector u, such that $\hat{u}\approx u$. More specifically, W and b denote the weight and bias matrices of the NN, respectively, and their values are the result of a training process [34], as well as for the parameter vector λ. Note that, since $\hat{u}$ is a function, its derivatives concerning the independent variable t can be computed during the training process through automatic differentiation (AD) [35,36]. Then, a function g outlining the approximation of Equation (1) can be defined, as reported in Equation (2):

(2) $$g={\hat{u}}^{\left(n\right)}-F(t,\hat{u},{\hat{u}}^{\prime },{\hat{u}}^{\prime \prime },\ldots ,{\hat{u}}^{\left(n-1\right)},\lambda )$$

**Figure 1:**
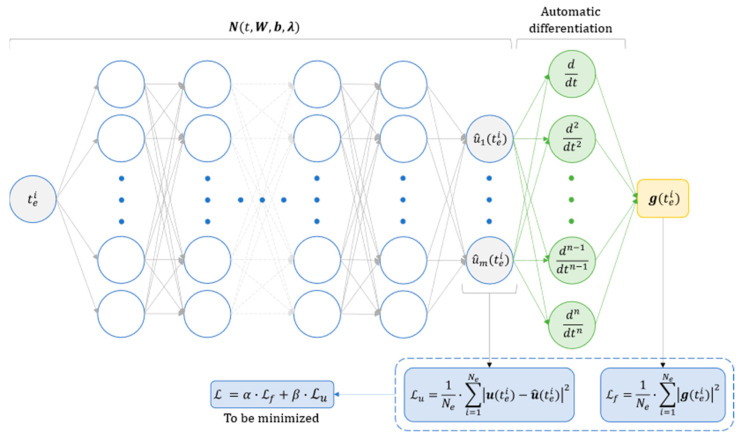
Scheme showing how a physics-informed neural network is trained to estimate the parameters vector λ of a system described by ODEs.

To enable the neural network to fine-tune the parameters W,b, and λ in order to (i) fulfil the underlying ODEs describing the system behaviour and (ii) to fit the available data (i.e., gathered measurements, in which the state vector u is known), two different loss functions are considered, as shown in the following Equations (3) and (4).

(3) $$L_{f}=\frac{1}{N_{e}}\cdot \sum_{i=1}^{N_{e}}{\left|g\left(t_{e}^{i}\right)\right|}^{2}$$

(4) $$L_{u}=\frac{1}{N_{e}}\cdot \sum_{i=1}^{N_{e}}{\left|u\left(t_{e}^{i}\right)-\hat{u}(t_{e}^{i})\right|}^{2}$$

where $L_{f}$ denotes the loss for the ODEs fulfilling while $L_{u}$ is the loss of the observed data; $t_{e}^{i}$ indicates the generic i-th element of the vector $t_{e}$, made of $N_{f}$ elements inside the domain $\left[t_{0},t_{f}\right]$, in which $L_{f}$ and $L_{u}$ are evaluated. Specifically, the acquisition time of the measured state vector u is typically employed as the vector $t_{e}$. These loss functions are subsequently integrated to yield the loss term L, as presented in Equation (5):

(5) $$L{=\alpha \cdot L}_{f}+{\beta \cdot L}_{u}$$

where α and β denote two coefficients employed to assign greater weight either to the contributions derived from the accessible data or those related to the system physics. Consequently, the objective of minimizing L is enforced, enabling the PINN to infer the unidentified parameters that define the system dynamics. Notably, the appropriate values for the coefficients α and β are determined through an iterative trial-and-error process. A scheme showing how the PINN is trained is presented in Figure 1. The input of the PINN is represented by the generic time instant $t_{e}^{i}$, and its output is the corresponding approximation of the components of the measured state vector u. In each training iteration, the PINN output is compared with the actual value of the components of the state vector for all the time instants $t_{e}^{i}$ within the vector $t_{e}$, resulting in the loss term $L_{u}$. Simultaneously, the PINN outputs are differentiated automatically to obtain the various terms of the n-th order ODEs described in Equation (1). This process enables the derivation of the residual g of the physics equation, from which the loss term $L_{f}$ is computed. The two losses are then combined to form the total loss term L, which is the metric to be minimized. Note that the hyperparameters of the PINN to be optimized are not only the weights W and biases b but also, and significantly, the parameter vector λ describing the system health state.

## 3. Case Study

### 3.1. Extended Jeffcott Rotor with Unknown System Parameters

**Figure 2:**
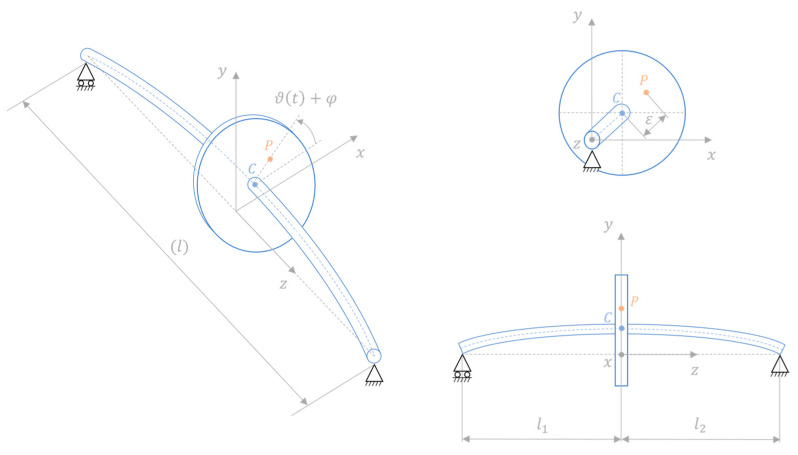
Scheme of the considered rotating shaft system with the disk geometrical centre (point C) and static unbalance (point P) highlighted.

The PINN approach described in the Section 2 is here applied on a numerical case study of a rotating shaft system in which the dynamics are simulated with the use of an extended Jeffcott rotor model [37]. The system, illustrated in Figure 2, consists of a 1 m long rotating shaft (i.e., l=1 m) made of aluminium (Young’s modulus E set to 70,000 MPa, and density ρ equal to 2700 kg·m^−3^) and supported at both ends. It has a circular cross section with a diameter of 20 mm, and it incorporates a disk (representing, for instance, a flywheel, fan, turbine, gear, etc.) that is mounted at distances $l_{1}$ and $l_{2}$ from the respective supports. The shaft rotates at a velocity $\Omega =\frac{d\vartheta (t)}{dt}$, in which ϑ(t) denotes the angle defined with respect to the x axis of the right-handed xyz reference frame which is fixed in space. In this reference frame, the disk lies within the xy plane, and the z axis is aligned with the line connecting the two supports. Without any loss of generality, the disk is here considered to be positioned at the midpoint of the shaft, i.e., $l_{1}=l_{2}=l/2$. Moreover, the disk centre of mass P is displaced from the axis of rotation, whose trace in the disk plane is identified with the point C, generating a static unbalance defined by the distance ε and the angle φ=ϑ(0). This unbalance causes the point C to displace from the line joining the supports leading the shaft to whirl around it.

The numerical model used to compute the rotor dynamics consists of a system of two second-order ODEs with a state vector of two components (making reference to Section 2, n=2 and p=2, respectively), as reported in Equations (6) and (7):

(6) $$\left[\begin{array}{cc} m & 0\\ 0 & m \end{array}\right]\left\{\begin{array}{c} {\ddot{x}}_{c}\\ {\ddot{y}}_{c} \end{array}\right\}+\left[\begin{array}{cc} c_{n}+c_{r} & 0\\ 0 & c_{n}+c_{r} \end{array}\right]\left\{\begin{array}{c} {\dot{x}}_{c}\\ {\dot{y}}_{c} \end{array}\right\}+\left[\begin{array}{cc} k_{x} & {\Omega \cdot c}_{r}\\ -\Omega {\cdot c}_{r} & k_{y} \end{array}\right]\left\{\begin{array}{c} x_{c}\\ y_{c} \end{array}\right\}=\left\{\begin{array}{c} f_{x}\\ f_{y} \end{array}\right\}$$

(7) $$\left\{\begin{array}{c} f_{x}\\ f_{y} \end{array}\right\}=\left\{\begin{array}{c} m_{d}\cdot \varepsilon \cdot \left(\Omega^{2}\cdot cos\left(\Omega \cdot t+\varphi \right)+\dot{\Omega }\cdot sin\left(\Omega \cdot t+\varphi \right)\right)\\ m_{d}\cdot \varepsilon \cdot \left(\Omega^{2}\cdot sin\left(\Omega \cdot t+\varphi \right)-\dot{\Omega }\cdot cos\left(\Omega \cdot t+\varphi \right)\right)-m\cdot g \end{array}\right\}$$

where $x_{c}$ and $y_{c}$ denote the x and y coordinates of the point C, respectively, and represent the components of the state vector (i.e., $u=[x_{c},y_{c}]$); $c_{n}$ and $c_{r}$ are the equivalent viscous damping terms of the stationary and rotating parts of the system, respectively; m represents the summation of the disk mass $m_{d}$ and the shaft equivalent mass at the disk location $m_{s}^{e}$; g indicates the gravity acceleration; $k_{x}$ and $k_{y}$ are the system stiffnesses along the x and y axes, respectively, that are assumed to coincide with the axes of the ellipse of elasticity, i.e., the principal axes of elasticity of the supporting structure. Notably, the rotating damping $c_{r}$ is here considered to be coincident to the contribution given by the shaft material properties, thus neglecting any potential additional term, and it is computed exploiting the approximation of a linear system [37], i.e., $c_{r}=2\xi_{r}\sqrt{k_{s}\cdot m_{s}}$. Here, $\xi_{r}$ denotes the rotational damping coefficient, that is assumed to be 0.001, $m_{s}$ represents the mass of the shaft, while $k_{s}=\frac{48\cdot E\cdot I}{l^{3}}$ denotes the shaft flexural stiffness, with I indicating the moment of inertia of the shaft cross section. The disk mass $m_{d}$ is considered to be equal to 2 kg, while the shaft equivalent mass at the disk location $m_{s}^{e}$ is computed as $m_{s}^{e}=\frac{k_{s}\cdot \delta }{g}$, in which $\delta =\frac{5\cdot m_{s}\cdot g\cdot l^{3}}{384\cdot E\cdot I}$ denotes the static displacement of the shaft at the disk location due to the shaft weight. The stiffnesses $k_{x}$ and $k_{y}$ are the combination of the shaft stiffness $k_{s}$ and those of the supports $k_{b}^{x}$ and $k_{b}^{y}$ along the x and y axes, respectively. That is, $\frac{1}{k_{x}}=\frac{1}{k_{s}}+\frac{1}{k_{b}^{x}}$ and $\frac{1}{k_{y}}=\frac{1}{k_{s}}+\frac{1}{k_{b}^{y}}$. However, determining the stiffness values of the supports can be challenging for different reasons, e.g., when they have complex geometries and interactions, due to misalignments or imperfections in installation, when the support materials are not well defined or uniform, and they can change due to degradation over time [38]. A similar reasoning applies for the non-rotating damping and for the static unbalance. Hence, in this work, ε, φ, $c_{n}$, $k_{x}$, and $k_{y}$ represent the components of the parameter vector λ, and their values are estimated through a PINN. Such parameters are selected because tracking their value is essential for maintaining the performance, reliability, and safety of rotating machinery.

**Figure 3:**
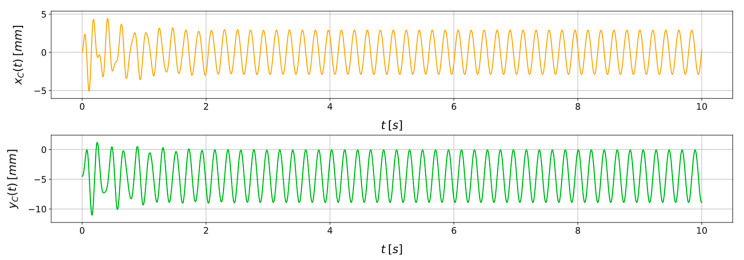
Example of the model responses $x_{C}$ and $y_{C}$ in a representative scenario with Ω=30 rad·s^−1^, ε=8.00 mm, φ=10.00 deg, $c_{n}=7.00\times 10^{-3}$ N·s·mm^−1^, $k_{x}=7.76$ N·mm^−1^, and $k_{y}=6.71$ N·mm^−1^, without adding artificial noise.

**Figure 4:**
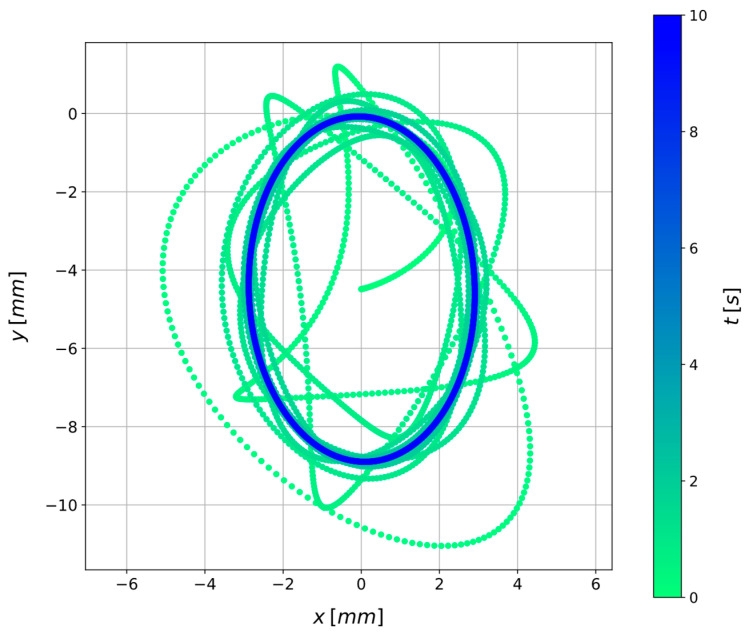
Visualization in the x−y plane of the disk centre position ($x_{C},y_{C}$) over time t for a scenario in which a constant rotational speed of Ω=30 rad·s^−1^ is imposed and where ε=8.00 mm, φ=10.00 deg, $c_{n}=7.00\times 10^{-3}$ N·s·mm^−1^, $k_{x}=7.76$ N·mm^−1^, $k_{y}=6.71$ N·mm^−1^, without adding artificial noise.

Figure 3 shows the model responses $x_{c}$ and $y_{c}$ obtained by solving Equations (6) and (7) with a Runge-Kutta 4/5 integration method [39] in the time range $\left[0,10\right]\;s$. In the simulated scenario, a constant rotational speed of Ω=30 rad·s^−1^ is imposed, and $k_{x}=7.76$ N·mm^−1^, $k_{y}=6.71$ N·mm^−1^, $c_{n}=7.0\times 10^{-3}$ N·s·mm^−1^, ε=8 mm, φ=10 deg. No artificial noise is added. Figure 4 shows the scatter plot of the position of the disk centre C in the xy plane over time.

### 3.2. System State Characterization through Physics-Informed Neural Networks

A PINN is then exploited to estimate the unknown parameters of the analysed rotating shaft system, i.e., to estimate the parameter vector $\lambda =[\varepsilon ,\varphi ,c_{n},k_{x},k_{y}]$. Notably, the employed NN architecture consists of 1 input neuron that takes in the generic time instant t, 1 hidden layer made of 200 neurons, and 2 output neurons to predict the values of $x_{C}$ and $y_{C}$. Moreover, the hyperbolic tangent tanh activation function [40] is used in the hidden layer, while the output layer embeds a linear activation function. The true system responses $x_{C}$ and $y_{C}$ required for training the PINN are numerically obtained with the Runge-Kutta 4/5 integration method in the time range [0,1] s (i.e., $t_{0}=0$ s and $t_{f}=1$ s). Within this range, the true solution is sampled with a sampling frequency of 10 kHz, which means that 10,001 equally spaced points are considered in time. The same time instants are also considered for building the vector $t_{e}$ that is used for training (i.e., $N_{f}=10,001$). It is worth noting that various sampling frequency values were examined in this study. Specifically, the investigation covered a sampling frequency range of [1, 10] kHz with increments of 3 kHz. Due to the similarity in outcomes from the analysis, these results are omitted here for the sake of conciseness.

**Figure 5:**
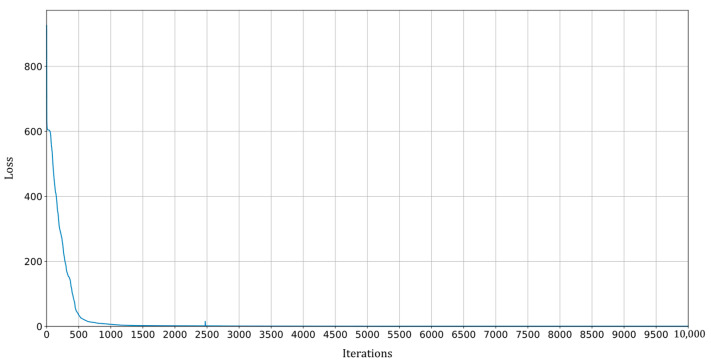
PINN training loss over the training iterations for a scenario in which a constant rotational speed of Ω=45 rad·s^−1^ is imposed and where ε=8.00 mm, φ=10.00 deg, $c_{n}=7.00\times 10^{-3}$ N·s·mm^−1^, $k_{x}=5.53$ N·mm^−1^, $k_{y}=7.25$ N·mm^−1^, without adding artificial noise.

**Figure 6:**
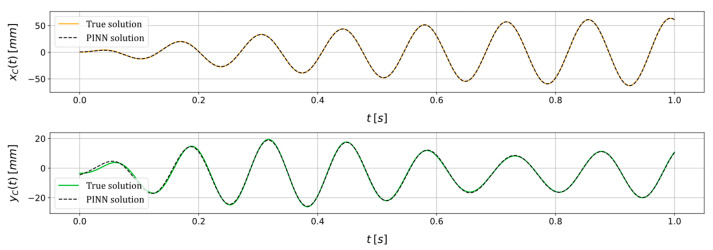
True and PINN solutions of the state variables $x_{C}$ and $y_{C}$ for a scenario in which a constant rotational speed of Ω=45 rad·s^−1^ is imposed and where ε=8.00 mm, φ=10.00 deg, $c_{n}=7.00\times 10^{-3}$ N·s·mm^−1^, $k_{x}=5.53$ N·mm^−1^, $k_{y}=7.25$ N·mm^−1^, without adding artificial noise.

A representative scenario is examined to assess the efficacy of the proposed methodology. The scenario involves the imposition of a constant rotational speed of Ω=45 rad·s^−1^, alongside the following unknown parameters: ε=8.00 mm, φ=10.00 deg, $c_{n}=7.00\times 10^{-3}$ N·s·mm^−1^, $k_{x}=5.53$ N·mm^−1^, $k_{y}=7.25$ N·mm^−1^. No artificial noise is added to the system responses. The PINN is trained on an AMD Ryzen 9 5900HX 3.30 GHz processor using a limited-memory Broyden–Fletcher–Goldfarb–Shanno (LBFGS) optimization algorithm [41]. The learning rate is set to η=0.01, while both the coefficients of the loss, α and β, are fixed at 1. The training process comprises 10,000 iterations. Moreover, the arbitrary initial guess set for the unknown parameters is ε=0.00 mm, φ=0.00 deg, $c_{n}=0.001\times 10^{-3}$ N·s·mm^−1^, $k_{x}=1.00$ N·mm^−1^, $k_{y}=2.00$ N·mm^−1^. The training process, as depicted in Figure 5, illustrates the progressive reduction of the training loss as the number of iterations increases. Instead, Figure 6 shows how the trained PINN is able to fit the available data to estimate the unknown parameters, showing a comparison between the true solution and the one obtained with the PINN. The outcome reveals that the approximation of the state vector offered by the PINN closely aligns with the actual vector, showcasing marginal disparities primarily observed in the state variable $y_{C}$ during the initial time instants. Finally, the true value and the correspondent PINN estimation for all the system unknown parameters is shown in Table 1. What emerges is that all the parameters are estimated by the PINN with a remarkable accuracy, presenting the best performance in identifying the system stiffness values $k_{x}$ and $k_{y}$, where the relative error in the estimation remains below 0.70%. Notably, among the parameters under estimation, the angle φ of the static unbalance and the non-rotating damping $c_{n}$ prove to be the most challenging. This observation finds potential justification in the relatively subdued impact these parameters exert on the state variables compared to their counterparts. Nevertheless, even in these instances, the relative error remains constrained within 5.80%.

**Figure 7:**
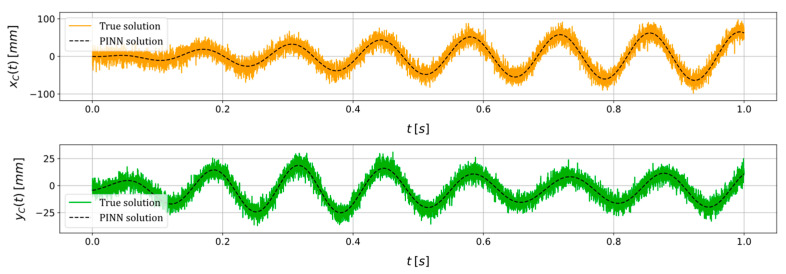
True and PINN solutions of the state variables $x_{C}$ and $y_{C}$ for a scenario in which a constant rotational speed of Ω=45 rad·s^−1^ is imposed and where ε=8.00 mm, φ=10.00 deg, $c_{n}=7.00\times 10^{-3}$ N·s·mm^−1^, $k_{x}=5.53$ N·mm^−1^, $k_{y}=7.25$ N·mm^−1^, adding an artificial noise with a SNR=20 dB.

In order to assess the robustness of the PINN to external influences affecting input data (e.g., measurement noise, environmental vibrations, etc.), the same scenario is revisited while maintaining consistent training process conditions (i.e., algorithm used, learning rate, initial parameters guess, etc.). In this context, the input variables $x_{C}$ and $y_{C}$, employed for the estimation of system parameters, are subjected to a perturbation through the introduction of supplementary numerical noise with a signal-to-noise ratio (SNR) of 20 dB. The data fitting performed by the PINN and the parameters estimation are reported in Figure 7 and Table 2, respectively. The PINN approximation of the state variables $x_{C}$ and $y_{C}$ appears to closely match the true solution. That is, the PINN manages to smooth out all the perturbances introduced by the added numerical noise, thus acting as a filter. The outcomes demonstrate that the PINN continues to function as a dependable tool for characterizing the health state of the system, even when confronted with external disturbances within the input data. Notably, no indications of a significant diminished algorithm performance are discernible, with all parameter estimations retaining a relative error of less than 8.00%.

**Figure 8:**
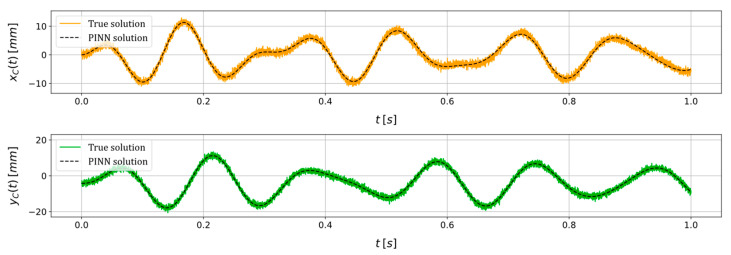
True and PINN solutions of the state variables $x_{C}$ and $y_{C}$ for a scenario in which a constant rotational speed of Ω=35 rad·s^−1^ is imposed and where ε=12.00 mm, φ=20.00 deg, $c_{n}=5.00\times 10^{-3}$ N·s·mm^−1^, $k_{x}=7.76$ N·mm^−1^, $k_{y}=6.14$ N·mm^−1^, adding an artificial noise with an SNR=30 dB.

Lastly, an additional example is presented below to assess the estimation capabilities of the PINN when fed with data pertaining to a scenario marked by distinct imposed conditions and health states. The scenario encompasses the application of a constant rotational speed of Ω=35 rad·s^−1^, accompanied by the following unknown parameters: ε=12.00 mm, φ=20.00 deg, $c_{n}=5.00\times 10^{-3}$ N·s·mm^−1^, $k_{x}=7.76$ N·mm^−1^, $k_{y}=6.14$ N·mm^−1^. Moreover, an artificial noise with an SNR of 30 dB is added to the input data, and the same training conditions of the previous scenarios are kept. The true and PINN solutions of the state variables $x_{C}$ and $y_{C}$ are reported in Figure 8, and the estimation of the unknown parameters is shown in Table 3. As for the previous scenario, the PINN approximation of the state variables $x_{C}$ and $y_{C}$ shows a strong correspondence with the true solution. The unknown parameters are satisfactorily estimated even in this scenario. The lowest relative estimation error characterizes the system stiffnesses $k_{x}$ and $k_{y}$, i.e., 0.52% and 0.16%, respectively, while the non-rotating damping $c_{n}$ is identified with a 9.80% error. 

### 3.3. Comparison with Traditional Optimization Algorithms for Parameters Estimation

The potentialities and limitations of the proposed framework are then identified by comparing its performance to that of traditional optimization algorithms. To this purpose, a gradient-based optimization algorithm [42] and a genetic algorithm [43] are employed to estimate the parameter vector $\lambda =[\varepsilon ,\varphi ,c_{n},k_{x},k_{y}]$. The gradient-based optimization algorithm leverages the *fmincon* nonlinear solver implemented in MATLAB. The optimization problem searches the target parameters within prescribed bounds, and a step tolerance of $1\times 10^{-10}$ is used for improved performance. Instead, the genetic algorithm used for estimating the unknown parameters is based on the *ga* MATLAB function. Population size of 200 and elite count of 2 are selected though trial and error. Crossover is applied through the built-in function *crossoverscattered*, while the selected mutation function is *mutationadaptfeasible*. Optimization is stopped according to the default early stopping criteria, or when 2000 generations are reached. Regardless of the optimzation algorithm employed, the loss function used to drive the optimization process involves the solution of the ODEs in Equations (6) and (7) to minimize the error between the computed and the observed displacement time history. As done for the PINN, the ODEs are solved using the Runge-Kutta 4/5 integration method.

First, the representative scenario involving the imposition of a constant rotational speed of Ω=45 rad·s^−1^, alongside the following unknown parameters: ε=8.00 mm, φ=10.00 deg, $c_{n}=7.00\times 10^{-3}$ N·s·mm^−1^, $k_{x}=5.53$ N·mm^−1^, and $k_{y}=7.25$ N·mm^−1^ is analysed. The results are shown in Table 4. Parameter φ is estimated with less accuracy than the PINN estimate shown in Table 1, while the accuracy is preserved for all the other variables. However, the optimization algorithms are much faster than the neural network-based framework, and the gradient-based algorithm allows for real-time estimation.

The parameters are then estimated in the case of noise with SNR of 20 dB affecting the input variables $x_{C}$ and $y_{C}$. The results are shown in Table 5. Similar considerations to those already reported above regarding the same scenario, but unaffected by noise, can be drawn out.

Finally, the last scenario presented in Section 3.2 is also analysed. That is, a constant rotational speed of Ω=35 rad·s^−1^ is considered, accompanied by the following unknown parameters: ε=12.00 mm, φ=20.00 deg, $c_{n}=5.00\times 10^{-3}$ N·s·mm^−1^, $k_{x}=7.76$ N·mm^−1^, $k_{y}=6.14$ N·mm^−1^. An artificial noise with an SNR of 30 dB is added to the input data. The results are shown in Table 6. The optimization algorithms perform similarly to the PINN-based framework in this scenario, with the advantage of allowing for real-time parameters estimation.

## 4. Conclusions

This paper has introduced a novel approach employing PINNs for estimating unknown parameters characterizing the health state of rotating shaft systems. The investigation has focused on a realistic numerical case study involving an extended Jeffcott rotor model, which has incorporated damping effects and anisotropic supports. The parameters considered have encompassed the radial and angular position of static unbalance caused by a shaft-mounted disk, stiffness values along the principal axes of elasticity, and the non-rotating damping coefficient. The estimation has relied exclusively on displacement signals from the disk centre, and various scenarios, incorporating different constant rotational speeds, have been thoroughly examined. Results have revealed the implemented PINN accuracy in estimating these parameters, demonstrating minimal relative errors even in the presence of substantial data noise. Moreover, the comparison with the estimates obtained using traditional optimization methods have revealed that PINNs slightly outperform gradient-based and genetic methods in terms of estimation accuracy, despite the longer processing time. Beyond optimizing machinery performance and enhancing efficiency and reliability, the proposed estimation method has facilitated predictive maintenance by early fault identification. 

The simulation experiments outlined in this paper establish a compelling proof of concept, showcasing the effectiveness of our proposed approach within a controlled environment. It is crucial to acknowledge that, while these simulations offer valuable insights, the next step involves experimental verification to ensure the real-world applicability of our methodology. Subsequent efforts will be dedicated to conducting experimental studies on more intricate case scenarios, aiming to provide a robust validation and refinement of our proposed approach.

## Figures and Tables

**Table 1:** True value and the correspondent PINN estimation for all the system unknown parameters related to a scenario in which a constant rotational speed of Ω=45 rad·s^−1^ is imposed; no noise added to the processed system responses.

Unknown Parameter	True Value	PINN Estimation	Relative Error (%)
ε [mm]	8.00	7.87	1.62
φ [deg]	10.00	9.42	5.80
$c_{n}$ [N·s·mm^−1^]	$7.00\times 10^{-3}$	$6.70\times 10^{-3}$	4.29
$k_{x}$ [N·mm^−1^]	5.53	5.54	0.18
$k_{y}$ [N·mm^−1^]	7.25	7.20	0.69

**Table 2:** True value and the correspondent PINN estimation for all the system unknown parameters related to a scenario in which a constant rotational speed of Ω=45 rad·s^−1^ is imposed; noise added to the processed system responses with an SNR=20 dB.

Unknown Parameter	True Value	PINN Estimation	Relative Error (%)
ε [mm]	8.00	7.91	1.12
φ [deg]	10.00	9.30	7.00
$c_{n}$ [N·s·mm^−1^]	$7.00\times 10^{-3}$	$6.45\times 10^{-3}$	7.86
$k_{x}$ [N·mm^−1^]	5.53	5.54	0.18
$k_{y}$ [N·mm^−1^]	7.25	7.29	0.55

**Table 3:** True value and the correspondent PINN estimation for all the system unknown parameters related to a scenario in which a constant rotational speed of Ω=35 rad·s^−1^ is imposed; noise added to the processed system responses with an SNR=30 dB.

Unknown Parameter	True Value	PINN Estimation	Relative Error (%)
ε [mm]	12.00	11.96	0.33
φ [deg]	20.00	21.17	5.85
$c_{n}$ [N·s·mm^−1^]	$5.00\times 10^{-3}$	$5.49\times 10^{-3}$	9.80
$k_{x}$ [N·mm^−1^]	7.76	7.72	0.52
$k_{y}$ [N·mm^−1^]	6.14	6.13	0.16

**Table 4:** True value and the correspondent optimization algorithms estimations related to a scenario in which a constant rotational speed of Ω=45 rad·s^−1^ is imposed; no noise added to the processed system responses.

Unknown Parameter	True Value	Gradient-Based Method		Genetic Algorithm	
		Estimation	Relative Error (%)	Estimation	Relative Error (%)
ε [mm]	8.00	8.00	0.00	7.60	5.00
φ [deg]	10.00	0.02	99.80	20.53	105.30
$c_{n}$ [N·s·mm^−1^]	$7.00\times 10^{-3}$	$6.09\times 10^{-3}$	13.00	$7.06\times 10^{-3}$	0.86
$k_{x}$ [N·mm^−1^]	5.53	5.66	2.35	5.39	2.53
$k_{y}$ [N·mm^−1^]	7.25	7.36	1.52	7.25	0.00

**Table 5:** True value and the correspondent optimization algorithms estimation related to a scenario in which a constant rotational speed of Ω=45 rad·s^−1^ is imposed; noise added to the processed system responses with an SNR=20 dB.

Unknown Parameter	True Value	Gradient-Based Method		Genetic Algorithm	
		Estimation	Relative Error (%)	Estimation	Relative Error (%)
ε [mm]	8.00	7.99	0.12	7.40	7.50
φ [deg]	10.00	0.01	99.90	1.92	80.80
$c_{n}$ [N·s·mm^−1^]	$7.00\times 10^{-3}$	$6.09\times 10^{-3}$	13.00	$4.84\times 10^{-3}$	30.86
$k_{x}$ [N·mm^−1^]	5.53	5.66	2.35	5.66	2.35
$k_{y}$ [N·mm^−1^]	7.25	7.35	1.38	7.30	0.69

**Table 6:** True value and the correspondent optimization algorithms estimation related to a scenario in which a constant rotational speed of Ω=35 rad·s^−1^ is imposed; noise added to the processed system responses with an SNR=30 dB.

Unknown Parameter	True Value	Gradient-Based Method		Genetic Algorithm	
		Estimation	Relative Error (%)	Estimation	Relative Error (%)
ε [mm]	12.00	11.8	1.67	10.2	15.00
φ [deg]	20.00	19.56	2.20	19.8	1.00
$c_{n}$ [N·s·mm^−1^]	$5.00\times 10^{-3}$	$5.41\times 10^{-3}$	8.20	$4.98\times 10^{-3}$	0.40
$k_{x}$ [N·mm^−1^]	7.76	7.76	0.00	7.53	2.96
$k_{y}$ [N·mm^−1^]	6.14	6.10	0.65	6.12	0.33

## Data Availability

The data presented in this study are available on request from the corresponding author.
